# Personalized treatment of women with early breast cancer: a risk-group specific cost-effectiveness analysis of adjuvant chemotherapy accounting for companion prognostic tests OncotypeDX and Adjuvant!Online

**DOI:** 10.1186/s12885-017-3603-z

**Published:** 2017-10-16

**Authors:** Beate Jahn, Ursula Rochau, Christina Kurzthaler, Michael Hubalek, Rebecca Miksad, Gaby Sroczynski, Mike Paulden, Marvin Bundo, David Stenehjem, Diana Brixner, Murray Krahn, Uwe Siebert

**Affiliations:** 1 Institute of Public Health, Medical Decision Making and Health Technology Assessment, Department of Public Health, Health Services Research and Health Technology Assessment, UMIT - University for Health Sciences, Medical Informatics and Technology, Eduard-Wallnöfer-Zentrum 1, A-6060 Hall i.T, Austria; 2 Division of Public Health Decision Modelling, Health Technology Assessment and Health Economics, ONCOTYROL - Center for Personalized Cancer Medicine, Karl-Kapferer-Straße 5, A-6020 Innsbruck, Austria; 3 Institut für Theoretische Physik, Universität Innsbruck, Technikerstraße 21A, A-6020 Innsbruck, Austria; 4 Department of Obstetrics and Gynecology, Medical University of Innsbruck, Christoph-Probst-Platz, Innrain 52, A-6020 Innsbruck, Austria; 5 Beth Israel Deaconess Medical Center, Harvard Medical School, 330 Brookline Ave, Boston, 02215 MA USA; 6 Toronto Health Economics and Technology Assessment (THETA) Collaborative, University of Toronto, Toronto General Hospital, 10EN, Room 249, 200 Elizabeth Street, Toronto, M5G 2C4 ON Canada; 7 Department of Emergency Medicine, University of Alberta, 116 St. and 85 Ave., Edmonton, AB T6G 2R3 Canada; 8 Department of Pharmacotherapy, University of Utah, 30 South 2000 East Room 4781, Salt Lake City, UT 84108 USA; 9 Huntsman Cancer Institute, University of Utah Hospitals & Clinics, 2000 Cir of Hope Dr, Salt Lake City, 84112 UT USA; 10 Program in Personalized Health, University of Utah, 15 North 2030 East, Room 2160, Salt Lake City, 84112 UT USA; 11 Center for Health Decision Science, Department of Health Policy and Management, Harvard T.H Chan School of Public Health, 718 Huntington Ave. 2nd Floor, Boston, 02115 MA USA; 12 Institute for Technology Assessment and Department of Radiology, Massachusetts General Hospital, Harvard Medical School, 101 Merrimac St., 10th FL, Boston, MA 02114 USA

**Keywords:** Cost-effectiveness analysis, Breast cancer, Adjuvant chemotherapy, Adjuvant!Online, OncotypeDX, Discrete event simulation, Personalized medicine, Decision analysis, Cost-utility analysis

## Abstract

**Background:**

Due to high survival rates and the relatively small benefit of adjuvant therapy, the application of personalized medicine (PM) through risk stratification is particularly beneficial in early breast cancer (BC) to avoid unnecessary harms from treatment. The new 21-gene assay (OncotypeDX, ODX) is a promising prognostic score for risk stratification that can be applied in conjunction with Adjuvant!Online (AO) to guide personalized chemotherapy decisions for early BC patients. Our goal was to evaluate risk-group specific cost effectiveness of adjuvant chemotherapy for women with early stage BC in Austria based on AO and ODX risk stratification.

**Methods:**

A previously validated discrete event simulation model was applied to a hypothetical cohort of 50-year-old women over a lifetime horizon. We simulated twelve risk groups derived from the joint application of ODX and AO and included respective additional costs. The primary outcomes of interest were life-years gained, quality-adjusted life-years (QALYs), costs and incremental cost-effectiveness (ICER). The robustness of results and decisions derived were tested in sensitivity analyses. A cross-country comparison of results was performed.

**Results:**

Chemotherapy is dominated (i.e., less effective and more costly) for patients with 1) low ODX risk independent of AO classification; and 2) low AO risk and intermediate ODX risk. For patients with an intermediate or high AO risk and an intermediate or high ODX risk, the ICER is below 15,000 EUR/QALY (potentially cost effective depending on the willingness-to-pay). Applying the AO risk classification alone would miss risk groups where chemotherapy is dominated and thus should not be considered. These results are sensitive to changes in the probabilities of distant recurrence but not to changes in the costs of chemotherapy or the ODX test.

**Conclusions:**

Based on our modeling study, chemotherapy is effective and cost effective for Austrian patients with an intermediate or high AO risk and an intermediate or high ODX risk. In other words, low ODX risk suggests chemotherapy should not be considered but low AO risk may benefit from chemotherapy if ODX risk is high. Our analysis suggests that risk-group specific cost-effectiveness analysis, which includes companion prognostic tests are essential in PM.

**Electronic supplementary material:**

The online version of this article (10.1186/s12885-017-3603-z) contains supplementary material, which is available to authorized users.

## Background

‘Personalized medicine’ (PM) is an increasingly relevant concept in clinical oncology. The term PM refers to an evolving approach to clinical decision making which seeks “to improve the stratification and timing of health care by utilizing biological information and biomarkers on the level of molecular disease pathways, genetics, proteomics as well as metabolomics” [1]. Although genomic information is considered to be the cornerstone of this discipline [2], clinical and sociodemographic characteristics of the patient and individual preferences can also be utilized to personalize medicine [3]. Because treatment strategies can be tailored in such a way that only patients who stand to benefit receive treatment [4], PM is particularly relevant in diseases, such as breast cancer, where in some cases the potential adverse effects may outweigh the benefits of treatment [5].

Breast cancer is among the most common types of cancer and a leading cause of cancer deaths in women. In Austria, breast cancer accounts for 30% of all tumors and for 16% of all cancer deaths in women [6]. The incidence of breast cancer in Austria in 2012 was about 76 cases per 100,000 women [6]. One of 13 females born in 2011 will develop breast cancer by the age of 75 years [7]. Aside from a small percentage of familial breast cancer, the risk factors for this malignancy are rather broad and vague: e.g., age, early menarche, late menopause, and obesity [8]. Although many treatment options are available [9, 10], the standard of care for early breast cancer is surgical resection, often followed by adjuvant radiation. Additional adjuvant systemic therapy depends on the hormone receptor status, such as estrogen receptor (ER) status, postmenopausal status, human epidermal growth factor receptor 2/neu (HER-2/neu) status, stage of the disease and co-morbidities.

For women with lymph node negative, estrogen receptor (ER) positive early-stage breast cancer who have relatively low recurrence risk adjuvant chemotherapy decision is complex and uncertain. While adjuvant systemic therapy can be beneficial for women at higher risk of a distant recurrence, it can cause more harm than benefit for low risk patients. Several prognostic tests are available to help identify women most likely to benefit from adjuvant systemic therapy in order to help guiding adjuvant therapy decision-making. For example, Adjuvant!Online is a free online tool that estimate risks and benefits of adjuvant therapy after breast cancer surgery based on factors, such as the patient’s stage, pathologic features, age and comorbidity level [11]. Mammaprint and OncotypeDX (ODX) are gene expression assays that “combine the measurements of gene expression levels within the tumor to produce a number associated with the risk of distant disease recurrence. These genetic tests aim to improve on risk stratification schemes based on clinical and pathologic factors currently used in clinical practice” [12]. As clinical decisions are increasingly based on the predictions of these tests, the additional costs should be considered in decision analyses of cancer management and treatment, similar to other companion diagnostics in PM [13].

Several studies have been conducted to evaluate the cost effectiveness or cost utility of different chemotherapy regimens. In our systematic literature search in CRD (Center for Reviews and Dissemination) [14], we found 24 cost-effectiveness studies that evaluate various chemotherapeutic regimens including capecitabine, cyclophosphamide, docetaxel, doxorubicin, epirubicin, eribulin, flourouracil, gemcitabine, ixabepilone, methotrexate, mytomycin, paclitaxel, vinblastine, and vinorebline. These studies were performed in the healthcare contexts of China, Canada, Germany, France, Italy, South Korea, Spain, UK, USA, The Netherlands, and Thailand. In particular, none were conducted for the Austrian healthcare context and none of these take into account personalized treatment decisions based on risk classification by AO and the new 21 gene assay ODX.

Our study focused on patients with ER and/or progesterone receptor (PR) positive, HER-2/neu negative and lymph node negative early breast cancer for whom AO and ODX risk classification may provide additional information that impacts decision-making. An advanced literature search conducted in PubMed [15] yielded no further studies on the combined prognostic approach of AO and ODX for these risk groups. Only Paulden et al. evaluate in a secondary analysis cost effectiveness of chemotherapy within risk groups according to AO and ODX. However, this was in a Canadian setting which differs from Austria (e.g., due to provided chemotherapy regimens and costs) [16].

The goal of the current study was to evaluate risk-group specific cost effectiveness of adjuvant chemotherapy for Austrian women with resected ER and/or PR positive, HER-2/neu negative, and lymph node negative early breast cancer. All potential risk groups according to the joint application of AO and ODX are considered. Additionally, we then compare these results to those from the Canadian study by Paulden et al. [16].

## Methods

### Modeling Framework

To analyze adjuvant test-treatment strategies for early breast cancer, we applied a decision-analytic computer simulation model [17] previously developed within our research center ONCOTYROL – Center for Personalized Cancer Medicine [18] (hereafter the “Oncotyrol breast cancer model”). The model validation and first application were recently published elsewhere [19, 20]. In this new model application, a hypothetical cohort of 50-year-old women diagnosed with ER and/or PR positive, HER-2/neu negative, lymph node negative breast cancer was simulated. We adopted a health care system perspective and lifetime horizon for this analysis. Outcomes of interests included survival (number of life years; LY), quality of life (number of quality-adjusted-life years; QALY), total costs (EUR) and incremental cost-effectiveness ratios (EUR/QALY). Costs and effects were discounted by 5% per year [21]. According to the ISPOR-SMDM guidelines [22], the model was implemented using a discrete event simulation approach (ARENA Version 13.90.00000, Rockwell Automation). This approach allows for individual patient pathways to be determined by multiple characteristics and test results, individual patient pathways to be recorded and time dependencies to be accounted for.

For reporting our modeling study, we followed the Consolidated Health Economic Evaluation Reporting Standards (CHEERS) Statement [23].

### Model structures

**Fig. 1:**
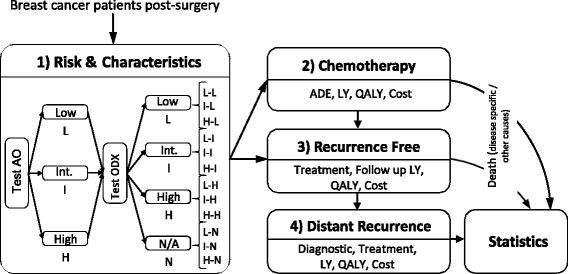
Schematic model structure. (Abbreviations: ADE-adverse drug event, LY-life years gained, QALY-quality adjusted-life years, AO-Adjuvant!Online, ODX-OncotypeDX), L-Low, Int./I-intermediate, H-High, combinations of risk classification (first letter representing Adjuvant!Online, second letter representing OncotypeDX: L-L, L-I, L-H, L-N, I-L, I-I, I-H, I-N, H-L, H-I, H-H, H-N). Source: adapted from Jahn et al. Lessons learned from a cross-model validation between a discrete event simulation model and a cohort state-transition model for personalized breast cancer treatment. Med Decis Making. 2016;36(3):375–390. Copyright © 2016 by Society for Medical Decision Making. Reprinted by permission of SAGE Publications, Inc.

The Oncotyrol breast cancer model is divided into different modules that describe the test-treatment strategies and the respective pathways of patients, their health states and key health events (Fig. 1).

In the beginning of the simulation (Module 1), patients enter the model, patient characteristics are assigned (age, time of death from other causes) and their AO risk score (individualized breast cancer specific mortality [BCSM]) and ODX risk classification (recurrence risk score [RS]) are calculated (BCSM: L ‘low’ BCSM < 9%, I ‘intermediate’ 9% ≤ BCSM < 17% or H ‘high’ BCSM ≥ 17% [24]; RS: L ‘low’ RS < 18, I ‘intermediate’ 18 ≤ RS < 30, H ‘high’ RS ≥ 30, N ‘RS not applied’). The costs and benefits of chemotherapy are quantified for each of the twelve combinations of risk classifications (where the first letter represents AO and second letter represents ODX: L-L, L-I, L-H, L-N, I-L, I-I, I-H, I-N, H-L, H-I, H-H, H-N). Two hypothetical cohorts were simulated in which all patients within these risk groups are assumed to receive or to not receive chemotherapy. Patients that pursue chemotherapy continue to Module 2 where chemotherapy and its associated adverse events (neutropenia, fever, infections, pain, nausea and gastrointestinal complications) are modeled. After chemotherapy, these patients are considered recurrence-free and are treated with aromatase inhibitors or tamoxifen for five subsequent years (Module 3). In addition, patients who do not receive chemotherapy enter Module 3 directly. Patients who face disease recurrence continue to Module 4 where further diagnostics and treatments are considered. We assume that patients with a distant recurrence remain in this health state and in Module 4 until they die from breast cancer. Throughout the entire simulated pathway, LYs, QALYs and costs are accumulated, and analyzed in the statistical module. In addition, all patients may die due to other causes at any time point and consequently leave the model.

### Model parameters

A detailed description of model parameters is provided elsewhere [19] and an overview of model parameters and sources are shown in Additional file 1: Table S1.

With respect to chemotherapeutic agents, we assumed all patients receive three cycles of FEC (5-fluorouracil, epirubicin, cyclophosphamide) followed by three cycles of DOC (docetaxel) [9]. After completion of adjuvant chemotherapy, all patients also received an aromatase inhibitor (anastozole, letrozole or exemestane) for five years. In cases in which no chemotherapy was provided, an aromatase inhibitor was started immediately.

Risk-group specific time to recurrence estimates were derived from Paulden et al. [16]. Treatment assumptions about distant recurrence were based on chart reviews by a senior gynecologist at Innsbruck Medical Hospital. The probability of death due to breast cancer in patients with distant recurrence was assumed to be identical in all patients regardless of the ER/PR status or the patient’s personal cancer history (median survival 25.8 months from time of diagnosis of recurrence [25]). Fatal toxicity of chemotherapy includes those patients who develop chemotherapy related acute myeloid leukemia (AML). All-cause mortality was applied throughout the entire simulated time horizon. Data were extrapolated using national life tables from Statistics Austria [26].

As ODX is currently not reimbursed in Austria, we relied on the manufacturer’s suggested retail price [27]. AO is available to medical experts free of charge [11]. We included direct costs for chemotherapy and related side effects (costs of chemotherapeutic agents, other supportive medications, such as pegfilgrastim and tropisetron, hospitalization, laboratory studies, and human resources), as well as costs of cancer follow-up, diagnosis and treatment of recurrent cancer [10, 25, 28] [Walter E: IPF, Vienna 2012, Report, unpublished]. Drug costs were based on pharmacy hospital prices. Utility weights were based on a recent cross-sectional observational study using the EuroQol five dimension questionnaire (EQ-5D) [29].

### Model validation

Model validation is a key modeling step for judging a model’s accuracy in making accurate predictions. Following the current ISPOR-SMDM best practice recommendations, the model was validated using face validation, internal validation and cross-model validation [30]. Further details are provided in Jahn et al. [20].

### Analysis

In the base-case analysis, we estimated discounted effects (LYs, QALYs) and costs of adjuvant chemotherapy in 12 different patient risk groups classified according to their AO (first letter) and ODX (second letter) risk classification (L-L, L-I, L-H, L-N, I-L, I-I, I-H, I-N, H-L, H-I, H-H, H-N). 100,000 patients were needed in the simulation in order to achieve stable results [20].

For each risk group, the simulation was run twice, the first assuming chemotherapy received by the patient and the second run assuming no chemotherapy received. The ICER was calculated by calculating the difference in discounted costs divided by the difference in discounted QALYs for these two alternatives. If one strategy is less effective but more expensive, then it is considered dominated and should not be considered. If chemotherapy is more effective but also more expensive, as compared to no chemotherapy, the ICER expresses the additional costs for one QALY gained. Chemotherapy is considered cost effective if the ratio is less than the willingness-to-pay (WTP) threshold.

As there is currently no explicit willingness-to-pay threshold for health technologies in Austria, we assumed a WTP of 50,000 EUR (alternatively 100,000 EUR) to test the robustness of our results and respective decisions in sensitivity analyses.

Parameter uncertainty was estimated using extensive deterministic one way sensitivity analyses on several parameters including age (40; 50; 70), discount rate (0; 2.5%; 5%), the cost of chemotherapy (+/− 10%), the cost of an ODX test set (+/− 10%), utilities (95% confidence intervals (CI) assuming a beta distribution), and the probability of distant recurrence (95% CI, assuming a beta distribution).

In a cross-country comparison, results were compared to the results of the Canadian modeling study by Paulden et al. [16] who applied a similar model structure. In contrast to our model, the Canadian model was designed as a probabilistic state-transition Markov [31] model for that particular health care setting which differ from Austria. For example, different chemotherapy regimens were considered (low risk patients: CMF (Cyclophosphamide, Methotrexate, 5-fluorouracil), intermediate risk patients: TC (Docetaxel, Cyclophosphamide), high risk patients: FEC-D 5-fluorouracil, Epirubicin, Cyclophosphamide, Docetaxel)). A list of parameter values for this model is provided in the Additional file 1. The modeling framework and the model structure are described elsewhere in greater detail [16].

## Results

### Base case

The results of the base-case analysis for the Austrian and the Canadian settings are displayed in Table 1. For each risk group, two lines depict the estimated, discounted LYs, QALYs and costs when chemotherapy is provided and when it is not. The ICER summarizes the results of chemotherapy or none.

**Table 1:** Discounted life-years, QALYs and incremental cost-effectiveness ratios of chemotherapy in the Austrian setting versus Canadian setting

			Austrian setting				Canadian setting			
Risk category		Chemo	LYs	QALYs	Costs (€)	ICER (€/QALY)	LYs	QALYs	Costs (€)	ICER (€/QALY)
AO	ODX									
Low	Low	No	15.51	12.04	11,021	D	15.36	11.93	10,088	D
		Yes	15.16	11.65	23,383		15.14	11.69	11,817	
Low	Int.	No	14.96	11.61	12,063	D	14.85	11.52	11,279	61,861
		Yes	15.05	11.57	23,608		15.04	11.61	16,562	
Low	High	No	12.06	9.31	17,520	3361	12.07	9.32	17,368	3118
		Yes	14.73	11.31	24,240		14.73	11.37	23,743	
Low	N/A	No	14.80	11.48	9230	566,277	14.69	11.40	8347	3768
		Yes	14.97	11.50	20,554		14.96	11.55	8925	
Int.	Low	No	15.21	11.80	11,557	D	15.10	11.72	10,708	D
		Yes	15.02	11.54	23,641		15.01	11.59	12,105	
Int.	Int.	No	13.71	10.62	14,421	13,504	13.69	10.60	13,872	4094
		Yes	14.77	11.34	24,163		14.78	11.40	17,150	
Int.	High	No	9.45	7.25	21,733	698	9.51	7.30	22,401	416
		Yes	14.61	11.21	24,500		14.60	11.26	24,049	
Int.	N/A	No	12.63	9.77	13,372	4790	12.63	9.76	12,927	548
		Yes	14.78	11.35	20,960		14.79	11.41	13,833	
High	Low	No	15.21	11.81	11,561	D	15.09	11.71	10,721	D
		Yes	15.03	11.55	23,666		15.01	11.59	12,116	
High	Int.	No	13.76	10.66	14,471	14,496	13.66	10.58	13,969	3940
		Yes	14.75	11.33	24,143		14.77	11.40	17,186	
High	High	No	9.43	7.23	21,815	676	9.48	7.27	22,474	400
		Yes	14.60	11.21	24,502		14.59	11.26	24,068	
High	N/A	No	12.09	9.34	14,257	3097	12.08	9.33	14,078	2816
		Yes	14.88	11.43	20,729		14.87	11.48	20,121	

*Abbreviations*: *AO* Adjuvant!Online, *ODX Oncotype*DX, *Int.* Intermediate, *LYs* life years, *QALYs* quality-adjusted life-years, *ICER* incremental cost-effectiveness ratio, *N/A* ODX test not applied

The results for the Austrian setting indicate that chemotherapy is dominated in the risk groups L-L (low AO, low ODX), L-I (low AO, intermediate ODX), I-L (intermediate AO, low ODX) and H-L (high AO, low ODX). Patients in these risk groups do not on average benefit from chemotherapy with respect to the clinical outcomes (LYs, QALYs). These results are consistent with the results for the Canadian setting with the exception of the L-I risk group (low AO and intermediate ODX).

In high risk ODX patients, chemotherapy seems to clearly be cost effective because an additional QALY can be gained at a low additional cost (ICER less than 3500 EUR/QALY). Chemotherapy is also cost effective in patients with an intermediate ODX risk and an intermediate or high AO risk chemotherapy with a WTP threshold of 15,000 EUR/QALY. These results are also consistent with the results from the Canadian setting. For patients in our model that are tested only with AO, chemotherapy is mainly cost effective with the exception of those who are AO low risk (L-N). These results differ slightly to the Canadian setting where chemotherapy for L-N patients is cost effective.

### Sensitivity analyses

Results of the sensitivity analyses are displayed in Table 2 (assuming WTP 50,000 EUR/QALY) and in the additional files (Additional file 2: Table S2A, Additional file 3: Table S2B, Additional file 4: Table S2C and Additional file 5: Table S2D assuming WTP 100,000 EUR/QALY). We ran the analysis for the four main risk groups (ODX low, ODX intermediate, ODX high, ODX not provided). In each block, we considered the respective AO risk in three columns. The first row in the table provides the results of the cost effectiveness of chemotherapy for each risk group in the base case. For example, for patients that have a low risk according to ODX and a low risk according to AO (L-L), chemotherapy was dominated (D) in the base case. In the following section, we display the results of the lower and upper bound when the parameters are varied. For example, we first consider a patient cohort age 40 (lower bound) and a patient cohort age 70 (upper bound). For the above risk group L-L, we observe that chemotherapy is still dominated, even if we vary the parameter age within the range of 40–70 years. We marked parameters depending on their impact on cost-effectiveness results and the following decisions: a) if the parameters that were changed led to the same decision based on the cost-effectiveness result, we use a white background, b) if those parameters that were varied led to a different decision based on the cost-effectiveness results, cells were colored with a dark grey. These were done assuming a WTP threshold of 50,000 EUR/QALY (Table 2) or 100,000 EUR/QALY (Additional file 2: Table S2A, Additional file 3: Table S2B, Additional file 4: Table S2C and Additional file 5: Table S2D).

**Table 2:**
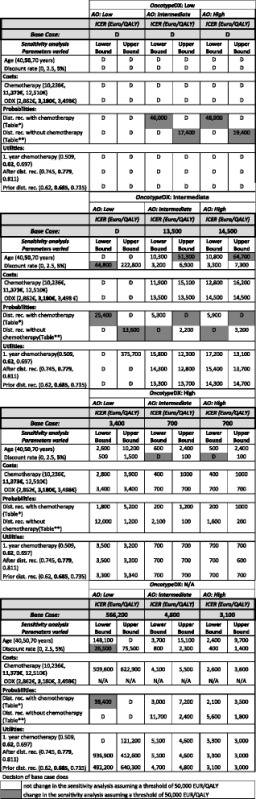
Sensitivity analyses of cost effectiveness of chemotherapy

*Abbreviations*: *AO* Adjuvant!Online, *D* dominated, *dist. rec.* distant recurrence, *N/A* not applied, bold numbers represent base case values

^a/b^base case ±2% for each risk group with/without chemotherapy, respectively

In summary, in one-way sensitivity analyses results were robust to changes in utilities, costs of chemotherapy and the genetic test ODX, a discount rate of 2.5% and patients at 40 years of age. For older age groups, the decision would be similar assuming a WTP of 65,000 EUR/QALY. The results, however, were sensitive to the probabilities of distant recurrence (with and without chemotherapy) especially within the risk groups L-I, I-L, I-I, H-L, H-I, L-N.

In the risk groups L-I, I-L and H-L, chemotherapy was dominated in the base case but was cost effective when the probabilities of distant recurrence were varied. In the risk groups I-I and H-I, chemotherapy was dominated when the probability of distant recurrence was varied. L-N became cost effective when the lower range of the probability of distant recurrence following chemotherapy was used.

## Discussion

In our cost-effectiveness analysis of adjuvant chemotherapy for early stage breast cancer patients, we evaluated upfront testing within the cancer management process similar to the evaluation of companion diagnostics. Our analysis shows that in the Austrian setting chemotherapy is effective and potentially cost effective for patients with an intermediate or high risk of disease according to ODX, independent from the AO risk classification (with the only exception of risk group L-I). In other words, low ODX risk suggests chemotherapy should not be considered but low AO risk may benefit from chemotherapy only if ODX risk is high.

Our results demonstrate that if the ODX test was not applied, chemotherapy would be considered cost effective for AO intermediate and high risk patients. However, based on the additional results of the ODX test, chemotherapy is dominated (less effective and more costly) for ODX low risk patients within these AO risk groups. Therefore, in the decision process we would not favor chemotherapy and consequently, reduce harms and costs. For low risk patients per the AO test, chemotherapy would very likely not be cost effective. However, after taking into account the additional information provided by the ODX test as well as the additional costs of this test, chemotherapy became cost effective for AO low and ODX high risk patients. In particular, these patients would greatly benefit from chemotherapy, both clinically and economically. In summary, our results demonstrate the importance of considering personalized information and additional costs in the evaluation of chemotherapy.

Sensitivity analyses demonstrate that the results are relatively robust with respect to the decisions about almost all model parameters except for the probability of distant recurrence within the risk groups L-I, I-L, I-I, H-L, H-I, L-N. For high risk patients per ODX and those classified only based on AO, the results are robust.

The advantage of our modeling approach is that, in addition to providing risk group-specific cost effectiveness of chemotherapy, we are also able to evaluate the effectiveness and cost effectiveness of the risk classification tools as previously shown [19]. Within this analysis, we considered that decisions regarding chemotherapy are based on the risk classification and additional factors. Therefore, only a percentage of patients would finally agree or not agree on chemotherapy in the respective risk groups. Our modular modeling structure approach allows one to adapt the model to evaluate additional test information or other innovative personalized test-treatment decisions.

Our results are consistent with the analysis of Paulden et al. [16] that showed a similar cost effectiveness of chemotherapy in the Canadian setting when using comparable risk classifications. In our systematic literature review, we identified no cost-effectiveness study for Austria nor any study that applied Adjuvant!Online or OncotypeDX. We identified four studies that sought to evaluate the same adjuvant chemotherapy regimen (FEC and TC). However, only one of these studies compared chemotherapy versus no chemotherapy. Campbell et al. [32] compared four strategies including one strategy without chemotherapy and three with different chemotherapy regimens. They found that “with an average to high risk of recurrence […], FEC-D appeared most cost effective assuming a threshold of £20,000 per QALY for the National Health Service (NHS). For younger low risk women, E-CMF (epirubicin, cyclophosphamide, methotrexate, fluorouracil) /FEC tended to be the optimal strategy and, for some older low risk women, the model suggested a policy of no chemotherapy was cost effective” [32]. These results were consistent with our results that also suggest that adjuvant chemotherapy is not cost effective in low-risk groups but is in high risk groups.

In Austria, there is currently no explicit threshold for health technologies to be considered cost effective. In other countries, thresholds vary and they are rarely disease or cancer specific. For example, in Canada, an oncology-specific ceiling threshold value of C$75,000 (equivalent to EUR 51,528) has been suggested and NICE (National Institute for Health and Care Excellence) provides a general threshold in 2012 of £18,317/QALY (EUR 23,180) that can be revised based on other factors [33].

Our study has several limitations. Although modeling studies allow information to be combined from different sources, we included as much Austrian data and local information on cancer management as possible. However, due to a lack of information about utility parameters and estimates for the risk of distant recurrence, we applied results from international studies. The underlying causes of hospitalizations were adapted for the Austrian context based on information of local clinical experts.

For some of the risk groups of interest, the decision regarding the provision of adjuvant chemotherapy may be predefined. However, we analyzed all potential groups for completeness. In addition to AO and ODX, there are other risk classification scores and genetic tests that may be used for this purpose. Since AO is continuously updated and free of charge, it was considered as first choice. Although not currently covered, ODX is a genetic test that has shown convincing analytical and clinical validity and therefore is likely to be implemented in clinical practice in Austria in the near future.

The ability to compare these results with the Canadian study results is limited due to the different health care settings (e.g., type of chemotherapy recommended, follow up treatment, cost structure), however, the results fall in a reassuringly similar direction.

In the future, our analysis could be applied in cost-effectiveness analyses based on risk classifications that are obtained from combinations of various multi-parameter molecular marker assays. At the fourteenth St. Gallen International Breast Cancer Conference, one expert panel discussed the role of multi-parameter molecular marker assays for prognosis and their value in selecting patients who require chemotherapy. “Oncotype DX®, MammaPrint®, PAM-50 ROR® score, EndoPredict® and the Breast Cancer Index® were all considered usefully prognostic for years 1-5” [34]. Beyond 5 years, reports suggest that these tests are prognostic [34]. The Panel agreed the PAM50 ROR® score to be clearly prognostic beyond 5 years. However, a clear majority rejected the prognostic value of MammaPrint®. For Oncotype DX®, the majority of the panel agreed with the potential value in predicting the usefulness of chemotherapy. Improved evidence supporting our modeling study will be provided by the TAILORx trail. After the full TAILORx trial results on ODX become available, we will rerun the analysis using updated input parameters including the probabilities of distant recurrence. Although there are promising alternative tests that allow personalized treatment decisions, multi-parameter molecular assays are expensive and may not be widely available [34].

Nevertheless, for reimbursement decisions, there have been strong efforts to enhance patient access to PM in Europe [35]. Decision-analytic modeling demonstrating cost effectiveness of combined test-treatment decisions may, therefore, provide particularly important information to decision makers and, potentially, improve accessibility to PM. For example, the ISPOR Personalized Medicine Special Interest Group notes that outcomes research and economic modeling can inform the assessment of PM at an early stage and supports prioritization of further research by early-stage decision modeling of potential cost-effectiveness and value of information (VOI) analyses [36]. Payne et al. derived recommendations to improve market access for companion diagnostics. Economic modeling is prescribed as a possible approach “to describe and quantify gaps in the evidence base and the added value of future research to reduce current uncertainties to support the introduction of companion diagnostics” [37].

The role of patient involvement has changed in recent years such that patients are increasingly included in the clinical decision making process. Therefore, personalized treatment has to account more actively for patient preferences and their individual state of health [4]. For decision-analytic modeling, as demonstrated in this study, future models should allow for patient-specific utility values. Further research in the field of companion diagnostics has identified an additional contribution of complementary diagnostics that goes beyond the usual health gains and cost savings. It highlights for example the value to the patient of having greater certainty of treatment benefit [38]. The upcoming publication of the EPEMED OHE study 2015 (European Personalized Medicine Association, Office of Health Economics) will provide insights on “how to articulate a value based evaluation per its economic, medical and full social appreciation and how a broader conception of value can be the path toward improving the HTA process” [39].

## Conclusion

Our decision analysis shows that in the Austrian setting, chemotherapy is usually effective and potentially cost effective for patients classified as intermediate or high risk according to ODX, independent from their AO risk classification. Without information from the genetic test ODX, chemotherapy would be assumed to be cost effective in intermediate and high risk patients per AO. However, there are specific risk groups (I-L, H-L) only identified by the genetic test that, on average, do not benefit from chemotherapy. Our analysis suggests that risk-group specific cost-effectiveness analyses that include the costs of companion diagnostics, including prognostic tests, are important in PM.

## Additional files

Additional file 1: Table S1. Model parameter overview. In the text of the manuscript, “Table S1” is referring to Table 1: “Model parameter overview”. Table 1 provides the set of input parameters that are used in the model. (DOCX 368 kb)

Additional file 2: Table S2A. Sensitivity Analysis of cost effectiveness of chemotherapy in subgroups with a low risk according to *Oncotype*DX. “Table S2A” is referring to Table 2a: “Sensitivity Analysis of cost effectiveness of chemotherapy in subgroups with a low risk according to *Oncotype*DX”. (DOCX 18 kb)

Additional file 3: Table S2B. Sensitivity Analysis of cost effectiveness of chemotherapy in subgroups with an intermediate risk according to *Oncotype*DX. “Table S2B” is referring to Table 2b: Sensitivity Analysis of cost effectiveness of chemotherapy in subgroups with an intermediate risk according to *Oncotype*DX. (DOCX 18 kb)

Additional file 4: Table S2C. Sensitivity Analysis of cost effectiveness of chemotherapy in subgroups with a high risk according to *Oncotype*DX. “Table S2C” is referring to Table 2c: Sensitivity Analysis of cost effectiveness of chemotherapy in subgroups with a high risk according to *Oncotype*DX. (DOCX 18 kb)

Additional file 5: Table S2D. Sensitivity Analysis of cost effectiveness of chemotherapy in subgroups where *Oncotype*DX is not applied. “Table S2D” is referring to Table 2d: Sensitivity Analysis of cost effectiveness of chemotherapy in subgroups where *Oncotype*DX is not applied. Table 2a, b, c and d show detailed results of the sensitivity analyses on the parameters age, discount rate, costs, probabilities and utilities. (DOCX 18 kb)

## Abbreviations

ADE: Adverse drug event
AML: Acute myeloid leukemia
AO: Adjuvant!Online
BC: Breast cancer
BCSM: Individualized breast cancer specific mortality
CHEERS: Consolidated Health Economic Evaluation Reporting Standards
CI: Confidence interval
CMF: Cyclophosphamide, methotrexate, 5-fluorouracil
CRD: Center for Reviews and Dissemination
D: Dominated
dist. rec.: Distant recurrence
DOC: Docetaxel
E-CMF: Epirubicin, cyclophosphamide, methotrexate and fluorouracil
EPEMED OHE: European Personalized Medicine Association, Office of Health Economics
EQ-5D: EuroQol five dimension questionnaire
ER: Estrogen receptor
EUR: Euro
FEC: 5-fluorouracil, epirubicin, cyclophosphamide
FEC-D: 5-fluorouracil, epirubicin, cyclophosphamide, docetaxel
FFG: Austrian Research Promotion Agency
H: High
HER-2/neu: Human epidermal growth factor receptor 2/neu
HTA: Health Technology Assessment
I, Int.: Intermediate
ICER: Incremental cost-effectiveness ratio
ISPOR: International Society for Pharmacoeconomics and Outcomes Research
L: Low
LY: Life years
N: Recurrence risk score not applied
N/A: Not applied
NHS: National Health Service
NICE: National Institute for Health and Care Excellence
ODX: OncotypeDX
PM: Personalized medicine
PR: Progesterone receptor
QALYs: Quality-adjusted life-years
RS: Recurrence risk score
SMDM: Society for Medical Decision Making
TC: Docetaxel, cyclophosphamide
VOI: Value of information
WTP: Willingness-to-pay
