# Supplementary material for: Personalized treatment of women with early breast cancer: a risk-group specific cost-effectiveness analysis of adjuvant chemotherapy accounting for companion prognostic tests OncotypeDX and Adjuvant!Online
Source: BMC Cancer. 2017 Oct 16;17:685. doi: 10.1186/s12885-017-3603-z (PMC5644100; doi:10.1186/s12885-017-3603-z)
Supplement: Supplementary file 1 — Model parameter overview. In the text of the manuscript, “Table S1” is referring to Table 1: “Model parameter overview”. Table 1 provides the set of input parameters that are used in the model. (DOCX 368 kb) [file 12885_2017_3603_MOESM1_ESM.docx]

Additional file 1: Table S1: Model parameter overview

| **Parameter** | **Base Case Oncotyrol M.** | **Base Case THETA M.** | **Source**  **Oncotyro M.** | **Source THETA M.** |
| --- | --- | --- | --- | --- |
| ***Probabilities*** | | | | |
| *Risk of hospital visit due to toxicity* | 17.04% | 17.04% | [1] | [2] |
| ***Cause of hospital visits due to toxicity*** | | | | |
| Neutropenia/fever/infections  Injuries & trauma  Malignant neoplasm  Pain & pain management  Nausea/vomiting/dehydration  Gastrointestinal tract  Chest pain | 53.56%  N/A  N/A  7.51%  6.02%  5.64%  N/A | 53.56%  11.48%  10.89%  7.51%  6.02%  5.64%  4.89% | [3] | [2] |
| ***Risk of 10 year distant recurrence without chemotherapy*** | | | | |
| Adjuvant! Online low risk  21-gene assay low risk  21-gene assay int. risk  21-gene assay high risk  Adjuvant! Online int. risk  21-gene assay low risk  21-gene assay int. risk  21-gene assay high risk  Adjuvant! Online high risk  21-gene assay low risk  21-gene assay int. risk  21-gene assay high risk | 6.75%  2.61%  5.78%  24.78%  20.60%  4.24%  13.40%  45.71%  24.12%  4.24%  13.40%  45.71% | 6.75%  2.61%  5.78%  24.78%  20.60%  4.24%  13.40%  45.71%  24.12%  4.24%  13.40%  45.71% | [4] | [5, 6] |
| ***Risk of 10 year distant recurrence with chemotherapy*** | | | | |
| Adjuvant! Online low risk  21-gene assay low risk  21-gene assay int. risk  21-gene assay high risk  Adjuvant! Online int. risk  21-gene assay low risk  21-gene assay int. risk  21-gene assay high risk  Adjuvant! Online high risk  21-gene assay low risk  21-gene assay int. risk  21-gene assay high risk | 4.93%  3.81%  4.46%  6.48%  6.07%  4.64%  6.23%  7.37%  7.68%  5.79%  8.18%  8.91% | 4.93%  3.81%  4.46%  6.48%  6.07%  4.64%  6.23%  7.37%  7.68%  5.79%  8.18%  8.91% | [4] | [5-11] |
| *Risk of mortality due to toxicity* | 0.1% | 0.35% | [3] | [12] |
| *Median life expectancy following distant recurrence (months)* | 25.8 | 21.0 | [3] | [13] |
| *Risk of mortality due to other causes* | Life Table Austria | Life Table Canada | [14] | [15] |
|  | | | | |
| ***Costs (inflated to 2011 Euros, Canadian Dollars)*** | | | | |
| 21-gene assay | 3,180€ | $4332.82 | [16] | [17, 18] |
| ***Costs for chemotherapy Austria*** | | | | |
| Echocardiography (one time) | 28€ | N/A | [1] | N/A |
| Chest radiography (one time) | 23€ |  |  |  |
| Port implantation (one time) | 550€ |  |  |  |
| Laboratory test (per cycle of chemotherapy) | 46.50€ |  |  |  |
| Blood panel (per week for 6 months) | 3.75€ |  |  |  |
| Human resources (per cycle of chemotherapy) | 48€ |  |  |  |
| Hospitalization (3 days) | 620€ |  |  |  |
| *Total additional costs for* *chemo* (6 months) | 2,089.50€ |  |  |  |
| *Total costs FEC*  (Fluorouracil 500mg/m^2^, Cyclophospamid 600mg/m^2^, Epirubicin 90mg/m^2^) | 672.16€ |  |  |  |
| *Total costs Taxotere*  (Docetaxel 75mg/m^2^) | 1,042.50€ |  |  |  |
| Neulesta (Pegfilgrastim) 6mg | 1,175.57€ |  |  |  |
| Navoban (Tropisetron-Hydrochlorid) 5mg 5 pills | 85.896€ |  |  |  |
| *Total costs for chemotherapy (Additional costs, FEC, Taxotere, Neulesta, Navoban, 6 months)* | 11,372.96€ |  | [1]*^,^*[3] |  |
| ***Costs for chemotherapy Canada*** | | | | |
| CMF  (Cyclophosphamide 600 mg/m^2^  Methotrexate 40 mg/m^2^  5-fluorouracil 600 mg/m^2^) | N/A | $949.84 | N/A | [19, 20] |
| FEC-D (5-fluorouracil 500 mg/m^2^, Epirubicin 100 mg/m^2^, Cyclophosphamide 500 mg/m^2^,  Docetaxel 100 mg/m^2^) |  | $15,544.78 |  |  |
| TC (Docetaxel 75 mg/m^2^, Cyclophosphamide 600 mg/m^2^) |  | $6,942.76 |  |  |
| ***Follow up Costs for the first 5 years after chemo therapy (costs per month/treatment)*** | | | | |
| Arimidex (Anastozol) 1mg | 73.77€ | N/A | [1] | N/A |
| Femara(Lertozolum) 2.5mg | *101.2€* |  |  |  |
| Aromasin (Exemestanum) 25mg | *75.87€* |  |  |  |
| Mammography | *32.00€* |  |  |  |
| Examination | *85.50€* |  |  |  |
| *Follow up costs per month* | *9.79€* | *$33.20* | *[1]^,^*[3] | [18] |
| *Follow up costs for the first 5 years after chemo per month* | *21.54€* | *$43.71* |  |  |
| *Other treatment costs for the first 5 years after chemo* | *5,016.80€* | $712.58 |  | [19] |
| ***Costs of diagnosing distant recurrence*** | | | | |
| Total costs of diagnosis of distant recurrence | *248.50€* | $8204.88 | *[1]*^,^[3] | [18] |
| ***Costs of treating distant recurrence*** | | | | |
| Total costs per 21 months | 32,015.26€ | $14,670.50 | [1] | [18] |
| End of life care (last 3 months ) | N/A | $21,627.42 |  |  |
| ***Treatment of non-fatal chemotherapy toxicity*** | | | | |
| Neutropenia/ fever/ infections  Injuries & trauma  Malignant neoplasm  Pain & pain management  Nausea/ vomiting/ dehydration  Gastrointestinal tract  Chest pain | 5,231.46€  N/A  N/A  3,270.66€  3,173.45€  5,169.31€  N/A | $6,699.29  $8,566.65  $6,628.00  $4,270.38  $4,064.37  $6,646.51  $2,966.04 | [21]^,^[3] | [21] |
| ***Treatment of fatal toxicity*** | 36,260.00€ | $33,173.36 | [22] | [21] |
|  | | | | |
| ***Utility weights*** | | | | |
| First year following diagnosis (while on hormone therapy) | 0.744 | 0.744 | [23] | [23] |
| First year following diagnosis (while on chemotherapy) | 0.620 | 0.620 |  |  |
| Second and following years prior to distant recurrence | 0.779 | 0.779 |  |  |
| Following distant recurrence | 0.685 | 0.685 |  |  |
| Dead | 0 | 0 |  |  |
|  | | | | |
| *** Parameter values differ for the two treatment strategies M1 and M2 | | | | |

Abbreviations: N/A – not applied

**References of table**

1. Tilak Financial Department: **Cost Data Report (unpublised)** In*.*; 2012.

2. **Available from:** [**http://www.cihi.ca**](http://www.cihi.ca) **[Accessed on March 18, 2013]**

3. Medical University Innsbruck: **Expert Opinion, Medical Record Review**. In*.* Innsbruck; 2012.

4. Paulden M, Franek J, Pham B, Bedard PL, Trudeau M, Krahn M: **Cost-Effectiveness of the 21-Gene Assay for Guiding Adjuvant Chemotherapy Decisions in Early Breast Cancer**. *Value in health : the journal of the International Society for Pharmacoeconomics and Outcomes Research* 2013, **16**:729-739.

5. Bryant J: **Toward a more rational selection of tailored adjuvant therapy. Data from the National Surgical Adjuvant Breast and Bowel Project. Presented at the primary therapy of early breast cancer**. In: *9th international conference January 26-28: 2005; St. Gallen, Switzerland*; 2005.

6. Paik S, Tang G, Shak S, Kim C, Baker J, Kim W, Cronin M, Baehner FL, Watson D, Bryant J *et al*: **Gene expression and benefit of chemotherapy in women with node-negative, estrogen receptor-positive breast cancer**. *Journal of clinical oncology : official journal of the American Society of Clinical Oncology* 2006, **24**(23):3726-3734.

7. Coombes RC, Bliss JM, Wils J, Morvan F, Espie M, Amadori D, Gambrosier P, Richards M, Aapro M, Villar-Grimalt A *et al*: **Adjuvant cyclophosphamide, methotrexate, and fluorouracil versus fluorouracil, epirubicin, and cyclophosphamide chemotherapy in premenopausal women with axillary node-positive operable breast cancer: results of a randomized trial. The International Collaborative Cancer Group**. *Journal of clinical oncology : official journal of the American Society of Clinical Oncology* 1996, **14**(1):35-45.

8. Fisher B, Brown AM, Dimitrov NV, Poisson R, Redmond C, Margolese RG, Bowman D, Wolmark N, Wickerham DL, Kardinal CG *et al*: **Two months of doxorubicin-cyclophosphamide with and without interval reinduction therapy compared with 6 months of cyclophosphamide, methotrexate, and fluorouracil in positive-node breast cancer patients with tamoxifen-nonresponsive tumors: results from the National Surgical Adjuvant Breast and Bowel Project B-15**. *Journal of clinical oncology : official journal of the American Society of Clinical Oncology* 1990, **8**(9):1483-1496.

9. French Adjuvant Study Group: **Benefit of a high-dose epirubicin regimen in adjuvant chemotherapy for node-positive breast cancer patients with poor prognostic factors: 5-year follow-up results of French Adjuvant Study Group 05 randomized trial**. *Journal of clinical oncology : official journal of the American Society of Clinical Oncology* 2001, **19**(3):602-611.

10. Jones S, Holmes FA, O'Shaughnessy J, Blum JL, Vukelja SJ, McIntyre KJ, Pippen JE, Bordelon JH, Kirby RL, Sandbach J *et al*: **Docetaxel With Cyclophosphamide Is Associated With an Overall Survival Benefit Compared With Doxorubicin and Cyclophosphamide: 7-Year Follow-Up of US Oncology Research Trial 9735**. *Journal of clinical oncology : official journal of the American Society of Clinical Oncology* 2009, **27**(8):1177-1183.

11. Roche H, Fumoleau P, Spielmann M, Canon JL, Delozier T, Serin D, Symann M, Kerbrat P, Soulie P, Eichler F *et al*: **Sequential adjuvant epirubicin-based and docetaxel chemotherapy for node-positive breast cancer patients: the FNCLCC PACS 01 Trial**. *Journal of clinical oncology : official journal of the American Society of Clinical Oncology* 2006, **24**(36):5664-5671.

12. Ludwig Breast Cancer Study Group: **Prolonged disease-free survival after one course of perioperative adjuvant chemotherapy for node-negative breast cancer**. *The New England journal of medicine* 1989, **320**(8):491-496.

13. Chang J, Clark GM, Allred DC, Mohsin S, Chamness G, Elledge RM: **Survival of patients with metastatic breast carcinoma: importance of prognostic markers of the primary tumor**. *Cancer* 2003, **97**(3):545-553.

14. **Statistiken - Demographische Maßzahlen: Sterbetafeln. Available from:** [**http://www.statistik.at/web_de/statistiken/menschen_und_gesellschaft/bevoelkerung/sterbetafeln/index.html**](http://www.statistik.at/web_de/statistiken/menschen_und_gesellschaft/bevoelkerung/sterbetafeln/index.html) **[Accessed 2012]**

15. **Complete life table, Ontario, 2000 to 2002: females, 2002. Available from:**[**http://www.statcan.gc.ca/pub/84-537-x/4064441-eng.htm**](http://www.statcan.gc.ca/pub/84-537-x/4064441-eng.htm) **[Accessed on March 18, 2013]**

16. Jahn B: **Personnel email-communication with manufacturer (unpublised)**. In*.*; 2012.

17. **Quaterly Report, 8 November 2011. Available from:** [**http://biz.yahoo.com/e/111108/ghdx10-q.html**](http://biz.yahoo.com/e/111108/ghdx10-q.html) **[Accessed on December 2, 2011]**

18. Will BP, Berthelot JM, Le Petit C, Tomiak EM, Verma S, Evans WK: **Estimates of the lifetime costs of breast cancer treatment in Canada**. *Eur J Cancer* 2000, **36**(6):724-735.

19. **Odette Cancer Centre. Available from:** [**http://sunnybrook.ca/content/?page=OCC_home**](http://sunnybrook.ca/content/?page=OCC_home) **[Accessed on March 18, 2013]**

20. Younis T, Rayson D, Sellon M, Skedgel C: **Adjuvant chemotherapy for breast cancer: a cost-utility analysis of FEC-D vs. FEC 100**. *Breast cancer research and treatment* 2008, **111**(2):261-267.

21. **Available from:** [**http://www.occp.com**](http://www.occp.com)**. [Accessed on March 18, 2013]**

22. Walter E: **IPF Institut für Pharmaökonomische Forschung, Report (unpublised)** In*.* Wien; 2012.

23. Lidgren M, Wilking N, Jonsson B, Rehnberg C: **Health related quality of life in different states of breast cancer**. *Quality of life research : an international journal of quality of life aspects of treatment, care and rehabilitation* 2007, **16**(6):1073-1081.
