# Supplementary material for: Personalized treatment of women with early breast cancer: a risk-group specific cost-effectiveness analysis of adjuvant chemotherapy accounting for companion prognostic tests OncotypeDX and Adjuvant!Online
Source: BMC Cancer. 2017 Oct 16;17:685. doi: 10.1186/s12885-017-3603-z (PMC5644100; doi:10.1186/s12885-017-3603-z)
Supplement: Supplementary file 2 — Sensitivity Analysis of cost effectiveness of chemotherapy in subgroups with a low risk according to OncotypeDX. “Table S2A” is referring to Table 2a: “Sensitivity Analysis of cost effectiveness of chemotherapy in subgroups with a low risk according to OncotypeDX”. (DOCX 18 kb) [file 12885_2017_3603_MOESM2_ESM.docx]

Additional file 2: Table S2A: Sensitivity Analysis of cost effectiveness of chemotherapy in subgroups with a low risk according to *Oncotype*DX

|  | |  | ***Oncotype*DX: Low** | | | | | | |
| --- | --- | --- | --- | --- | --- | --- | --- | --- | --- |
|  | |  | ***AO: Low*** | | ***AO: Intermediate*** | | | ***AO: High*** | |
|  | | | ***ICER (Euro/QALY)*** | | ***ICER (Euro/QALY)*** | | ***ICER (Euro/QALY)*** | | |
| ***Base Case Analysis:*** | | | **D** | | **D** | | **D** | | |
| ***Sensitivity analysis***  ***Parameters varied*** | | | **Lower Bound** | **Upper Bound** | **Lower Bound** | **Upper Bound** | **Lower Bound** | | **Upper Bound** |
| Age (40,**50**,70 years) | | | D | D | D | D | D | | D |
| Discount rate (0, 2.5, **5**%) | | | D | D | D | D | D | | D |
| **Costs:** | | | | | | | | | |
| Chemotherapy (10,236€, **11,373€**, 12,510€) | | | D | D | D | D | D | | D |
| ODX (2,862€, **3,180€,** 3,498€) | | | D | D | D | D | D | | D |
| **Probabilities:** | | | | | | | | | |
| Dist. rec. with chemotherapy (Table*) | | | D | D | 46,000 | D | 48,900 | | D |
| Dist. rec. without chemotherapy (Table**) | | | D | D | D | 17,400 | D | | 19,400 |
| **Utilities:** | | | | | | | | | |
| 1. year chemotherapy (0.509, **0.62**, 0.697) | | | D | D | D | D | D | | D |
| After dist. rec. (0.745, **0.779**, 0.811) | | | D | D | D | D | D | | D |
| Prior dist. rec. (0.62, **0.685**, 0.735) | | | D | D | D | D | D | | D |
| Decision in the base case analysis does | | | | | | | | | |
|  | not change in the sensitivity analysis assuming a threshold of 100,000 EUR/QALY | | | | | | | | |
|  | change in the sensitivity analysis assuming a threshold of 100,000 EUR/QALY | | | | | | | | |

*/** base case ± 2% for each risk group with/without chemotherapy, respectively; Abbreviations: AO – Adjuvant!Online, D – dominated, dist. rec. – distant recurrence, bold parameter numbers represent base case
