# Supplementary material for: Personalized treatment of women with early breast cancer: a risk-group specific cost-effectiveness analysis of adjuvant chemotherapy accounting for companion prognostic tests OncotypeDX and Adjuvant!Online
Source: BMC Cancer. 2017 Oct 16;17:685. doi: 10.1186/s12885-017-3603-z (PMC5644100; doi:10.1186/s12885-017-3603-z)
Supplement: Supplementary file 3 — Sensitivity Analysis of cost effectiveness of chemotherapy in subgroups with an intermediate risk according to OncotypeDX. “Table S2B” is referring to Table 2b: Sensitivity Analysis of cost effectiveness of chemotherapy in subgroups with an intermediate risk according to OncotypeDX. (DOCX 18 kb) [file 12885_2017_3603_MOESM3_ESM.docx]

Additional file 3: Table S2B: Sensitivity Analysis of cost effectiveness of chemotherapy in subgroups with an intermediate risk according to *Oncotype*DX

|  | | ***Oncotype*DX: Intermediate** | | | | | |
| --- | --- | --- | --- | --- | --- | --- | --- |
|  | | ***AO: Low*** | | ***AO: Intermediate*** | | ***AO: High*** | |
|  | | ***ICER (Euro/QALY)*** | | ***ICER (Euro/QALY)*** | | ***ICER (Euro/QALY)*** | |
| ***Base Case:*** | | **D** | | **13,500** | | **14,500** | |
| ***Sensitivity analysis***  ***Parameters varied*** | | **Lower Bound** | **Upper Bound** | **Lower Bound** | **Upper Bound** | **Lower Bound** | **Upper Bound** |
| Age (40,**50**,70 years) | | D | D | 10,300 | 51,300 | 10,800 | 64,700 |
| Discount rate (0, 2.5, **5**%) | | 44,800 | 222,800 | 3,200 | 6,900 | 3,300 | 7,300 |
| **Costs:** | | | | | | | |
| Chemotherapy (10,236€, **11,373€**, 12,510€) | | D | D | 11,900 | 15,100 | 12,800 | 16,200 |
| ODX (2,862€, **3,180€**, 3,498 €) | | D | D | 13,500 | 13,500 | 14,500 | 14,500 |
| **Probabilities:** | | | | | | | |
| Dist. rec. with chemotherapy (Table*) | | 25,400 | D | 5,300 | D | 5,900 | D |
| Dist. rec. without chemotherapy (Table**) | | D | 13,600 | D | 2,200 | D | 3,200 |
| **Utilities:** | | | | | | | |
| 1. year chemotherapy (0.509, **0.62**, 0.697) | | D | 375,700 | 15,800 | 12,300 | 17,200 | 13,100 |
| After dist. rec. (0.745, **0.779**, 0.811) | | D | D | 14,300 | 12,800 | 15,400 | 13,700 |
| Prior dist. rec. (0.62, **0.685**, 0.735) | | D | D | 13,300 | 13,700 | 14,300 | 14,700 |
| Decision in the base case analysis does | | | | | | | |
|  | not change in the sensitivity analysis assuming a threshold of 100,000 EUR/QALY | | | | | | |
|  | change in the sensitivity analysis assuming a threshold of 100,000 EUR/QALY | | | | | | |

*/** base case ± 2% for each risk group with/without chemotherapy, respectively; Abbreviations: AO – Adjuvant!Online, D – dominated, dist. rec. – distant recurrence, bold parameter numbers represent base case
