# Supplementary material for: Personalized treatment of women with early breast cancer: a risk-group specific cost-effectiveness analysis of adjuvant chemotherapy accounting for companion prognostic tests OncotypeDX and Adjuvant!Online
Source: BMC Cancer. 2017 Oct 16;17:685. doi: 10.1186/s12885-017-3603-z (PMC5644100; doi:10.1186/s12885-017-3603-z)
Supplement: Supplementary file 4 — Sensitivity Analysis of cost effectiveness of chemotherapy in subgroups with a high risk according to OncotypeDX. “Table S2C” is referring to Table 2c: Sensitivity Analysis of cost effectiveness of chemotherapy in subgroups with a high risk according to OncotypeDX. (DOCX 18 kb) [file 12885_2017_3603_MOESM4_ESM.docx]

Additional file 4: Table S2C: Sensitivity Analysis of cost effectiveness of chemotherapy in subgroups with a high risk according to *Oncotype*DX

|  | | ***Oncotype*DX: High** | | | | | | |
| --- | --- | --- | --- | --- | --- | --- | --- | --- |
|  | | ***AO: Low*** | | ***AO: Intermediate*** | | ***AO: High*** | | |
|  | | ***ICER (Euro/QALY)*** | | ***ICER (Euro/QALY)*** | | ***ICER (Euro/QALY)*** | | |
| ***Base Case:*** | | **3,400** | | **700** | | **700** | | |
| ***Sensitivity analysis***  ***Parameters varied*** | | **Lower Bound** | **Upper Bound** | **Lower Bound** | **Upper Bound** | **Lower Bound** | **Upper Bound** | |
| Age (40,**50**,70 years) | | 2,600 | 10,200 | 600 | 2,400 | 500 | 2,400 | |
| Discount rate (0, 2.5, **5**%) | | 500 | 1,500 | D | 100 | D | 100 | |
| **Costs:** | | | | | | | | |
| Chemotherapy (10,236€, **11,373€**, 12,510€) | | 2,800 | 3,900 | 400 | 1000 | 400 | 1000 | |
| ODX (2,862€, **3,180€**, 3,498 €) | | 3,400 | 3,400 | 700 | 700 | 700 | 700 | |
| **Probabilities:** | | | | | | | | |
| Dist. rec. with chemotherapy (Table*) | | 1,800 | 5,200 | 200 | 1,200 | 200 | 1000 | |
| Dist. rec. without chemotherapy (Table**) | | 12,000 | 1,200 | 2,100 | 100 | 1,600 | 200 | |
| **Utilities:** | | | | | | | | |
| 1. year chemotherapy (0.509, **0.62**, 0.697) | | 3,500 | 3,200 | 700 | 700 | 700 | | 700 |
| After dist. rec. (0.745, **0.779**, 0.811) | | 3,500 | 3,200 | 700 | 700 | 700 | | 600 |
| Prior dist. rec. (0.62, **0.685**, 0.735) | | 3,300 | 3,340 | 700 | 700 | 700 | | 700 |
| Decision in the base case analysis does | | | | | | | | |
|  | not change in the sensitivity analysis assuming a threshold of 100,000 EUR/QALY | | | | | | | |
|  | change in the sensitivity analysis assuming a threshold of 100,000 EUR/QALY | | | | | | | |

*/** base case ± 2% for each risk group with/without chemotherapy, respectively; Abbreviations: AO – Adjuvant!Online, D – dominated, dist. rec. – distant recurrence, bold parameter numbers represent base case
