# Supplementary material for: Personalized treatment of women with early breast cancer: a risk-group specific cost-effectiveness analysis of adjuvant chemotherapy accounting for companion prognostic tests OncotypeDX and Adjuvant!Online
Source: BMC Cancer. 2017 Oct 16;17:685. doi: 10.1186/s12885-017-3603-z (PMC5644100; doi:10.1186/s12885-017-3603-z)
Supplement: Supplementary file 5 — Sensitivity Analysis of cost effectiveness of chemotherapy in subgroups where OncotypeDX is not applied. “Table S2D” is referring to Table 2d: Sensitivity Analysis of cost effectiveness of chemotherapy in subgroups where OncotypeDX is not applied. Table 2a, b, c and d show detailed results of the sensitivity analyses on the parameters age, discount rate, costs, probabilities and utilities. (DOCX 18 kb) [file 12885_2017_3603_MOESM5_ESM.docx]

Additional file 5: Table S2D: Sensitivity Analysis of cost effectiveness of chemotherapy in subgroups where *Oncotype*DX is not applied

|  | | ***Oncotype*DX: N/A** | | | | | |
| --- | --- | --- | --- | --- | --- | --- | --- |
|  | | ***AO: Low*** | | ***AO: Intermediate*** | | ***AO: High*** | |
|  | | ***ICER (Euro/QALY)*** | | ***ICER (Euro/QALY)*** | | ***ICER (Euro/QALY)*** | |
| ***Base Case:*** | | **566,200** | | **4,800** | | **3,100** | |
| ***Sensitivity analysis***  ***Parameters varied*** | | **Lower Bound** | **Upper Bound** | **Lower Bound** | **Upper Bound** | **Lower Bound** | **Upper Bound** |
| Age (40,50,70 years) | | 148,100 | D | 3,700 | 15,100 | 2,400 | 9,700 |
| Discount rate (0, 2.5, 5%) | | 26,500 | 75,500 | 800 | 2,300 | 400 | 1,400 |
| **Costs:** | | | | | | | |
| Chemotherapy (10,236€, **11,373€**, 12,510€) | | 509,600 | 622,900 | 4,100 | 5,500 | 2,600 | 3,600 |
| ODX (2,862€, **3,180€**, 3,498 €) | | N/A | N/A | N/A | N/A | N/A | N/A |
| **Probabilities:** | | | | | | | |
| Dist. rec. with chemotherapy (Table*) | | 38,400 | D | 3,000 | 7,200 | 2,100 | 3,500 |
| Dist. rec. without chemotherapy (Table**) | | D | D | 11,700 | 2,400 | 5,600 | 1,800 |
| **Utilities:** | | | | | | | |
| 1. year chemotherapy (0.509, **0.62**, 0.697) | | D | 121,200 | 5,100 | 4,600 | 3,300 | 3,000 |
| After dist. rec. (0.745, **0.779**, 0.811) | | 936,900 | 412,600 | 5,100 | 4,600 | 3,300 | 3,000 |
| Prior dist. rec. (0.62, **0.685**, 0.735) | | 492,200 | 640,300 | 4,700 | 4,800 | 3,100 | 3,000 |
| Decision in the base case analysis does | | | | | | | |
|  | not change in the sensitivity analysis assuming a threshold of 100,000 EUR/QALY | | | | | | |
|  | change in the sensitivity analysis assuming a threshold of 100,000 EUR/QALY | | | | | | |

*/** base case ± 2% for each risk group with/without chemotherapy, respectively; Abbreviations: AO – Adjuvant!Online, D – dominated, dist. rec. – distant recurrence, N/A – not applied, bold parameter numbers represent base case
